# Inequality in Health Services for Internal Migrants in China: A National Cross-Sectional Study on the Role of Fund Location of Social Health Insurance

**DOI:** 10.3390/ijerph17176327

**Published:** 2020-08-31

**Authors:** Qiang Yao, Chaojie Liu, Ju Sun

**Affiliations:** 1 School of Political Science and Public Administration, Wuhan University, Wuhan 430072, China; yaoqiang@whu.edu.cn; 2 School of Psychology and Public Health, La Trobe University, Melbourne 3086, Australia; 3 Institute of Health, Wuhan University, Wuhan 430071, China

**Keywords:** health inequalities, migrants, social health insurance, China

## Abstract

On-the-spot settlements of medical bills for internal migrants enrolled with a social health insurance program outside of their residential location have been encouraged by the Chinese government, with the intention to improve equality in healthcare services. This study compared the use of health services between the internal migrants who had local health insurance coverage and those who did not. Data (n = 144,956) were obtained from the 2017 China Migrants Dynamic Survey. Use of health services was assessed by two indicators: visits to physicians when needed and registration (shown as health records) for essential public health services. Multi-level logistic regression models were established to estimate the effect size of fund location on the use of health services after controlling for variations in other variables. The respondents who enrolled with a social health insurance scheme locally were more likely to visit physicians when needed (adjusted odds ratio (AOR) = 1.18, 95% CI = 1.06–1.30) and to have a health record (AOR = 1.47, 95% CI = 1.30–1.65) compared with those who enrolled outside of their residential location: a gap of 3.5 percentage points (95% CI: 1.3%–5.8%) and 6.1 percentage point (95% CI: 4.3%–7.8%), respectively. The gaps were larger in the rural-to-urban migrants than those in the urban-to-urban migrants (AOR = 1.17, 95% CI = 0.93–1.48 for visiting physicians when needed; AOR = 0.71, 95% CI = 0.54–0.93 for having a health record). The on-the-spot medical bill settlement system has yet to fully achieve its proposed potential as inequalities in both medical and public health services remain between the internal migrants with and without local health insurance coverage. Further studies are needed to investigate how on-the-spot settlements of medical bills are implemented through coordination across multiple insurance funds.

## 1. Introduction

The term “internal migrants” refers to people who move to and live in a place outside of their registered residency as defined by the household registration system (also known as “Hukou”) in China [1,2]. Hukou was created in late 1950s in line with where a person was born [3,4]. In the early stage (1950s–1980s), it was designed to restrict population mobility, serving as an important infrastructure for resource allocations under the planned economy [3,5], especially for the dual (urban vs rural) social welfare systems [6]. For example, rural people were assigned with local farming lands and not allowed to be employed in an urban setting [4]. Meanwhile, public and social services such as job opportunities, housing allowance, educational opportunities, healthcare services, social insurance and other welfare entitlements were always linked to Hukou managed by local governments. Moving to a place without Hukou meant loss of all of the entitlements [1,7,8,9,10].

Since China adopted the market economic reform in the 1980s, the Hukou system has evolved to accommodate the increasing demand for urban workforces [3,4]. The attitudes of the Chinese government toward internal population mobility have shifted from “restriction” to “encouragement and assistance” [7,11]. People no longer worried about their subsistence needs without a Hukou [4,7]. As a result, a large number of rural workforces were freed up and started to seek jobs outside their Hukou location. The rapid industrialization, in particular in the eastern coastal region, attracted large scales of population internal migration [12]. The population of internal migrants increased from about 6 million in the 1980s to 244 million in 2017, accounting for about 18% of the population size nowadays in China [13,14]. The majority of internal migrants occupied job positions that would not otherwise be filled by the locals [15], making a great contribution to the economic development in China over the past few decades [16,17].

However, Hukou has continued to prevent internal migrants from enjoying the full benefits of economic and social development in China. On one hand, the fundamental link between Hukou and welfare entitlements remains. The great disparity in economic development across regions in China has been accompanied by increasing inequalities in health and welfare entitlements [18]. It is a common practice in the more developed regions to set up a separate social welfare system for internal migrants, which cannot match those for local Hukou holders [1,2,4]. On the other hand, it is very hard for internal migrants to change Hukou registration [3,4] unless they have obtained higher education and skills. In fact, the majority of internal migrants have moved from rural to urban, had low levels of education, taken up labor-intensive jobs with poor working environments, and lived in poor housing conditions [19]. The high health risks experienced by these internal migrants can be further exacerbated by their poor health literacy and a lack of access to the local welfare system. Several previous studies showed that internal migrants are at a high risk of occupational diseases, infectious and communicable diseases, sexual health problems, maternal health problems, and psychological problems [11,15]. In addition, they are more likely to abandon needed health services than their local counterparts, resulting in higher health loss and worse health status [11,15,20,21,22,23].

Similar to other social welfare systems, China has developed a Hukou-based social health insurance system, which comprises three schemes: Basic Medical Insurance for Urban Employees (BMIUE), Basic Medical Insurance for Urban Residents (BMIUR), and Rural New Cooperative Medical Scheme (RNCMS) [24,25,26]. While the BMIUE is co-funded by employer and employee contributions, the other two are heavily subsidized by local governments (including financial transfer from the central government). Under each scheme, there exist many funds administrated by municipal or county levels of government. There are great variations in these insurance policies. Even within a prefecture/county, urban–rural disparities are significant. The existence of thousands of social health insurance funds and a lack of coordination and coherent policies in China has created a great barrier for fund transfer and cross-fund settlements [27]. To maintain financial viability, these funds impose various restrictions on member benefits, such as restricted access to local providers (with a contract) and requirements for deductibles, co-payments and co-insurance. For example, each and every fund signs contracts with its own local providers for medical services delivery. Referral is required to obtain access to services provided by non-contracted providers [28]. Even with a referral, financial compensations for healthcare services outside of the designated region of the fund are always lower than those which are local [1,28,29]. Meanwhile, there are tedious paperwork requirements to obtain financial reimbursements [1,30]. This makes transfer of insurance enrollment across different funds and regions extremely challenging, if not impossible. Meanwhile, medical services outside of local contracted providers usually attract higher levels of out-of-pocket payments [1], jeopardizing the accessibility of healthcare services of internal migrants [11,13].

Although the vast majority of people in China have been covered by social health insurance, inequality in insurance coverage and entitlements has remained a serious policy concern. Studies showed that about 10% internal migrants failed to enroll in a social health insurance program due to the complexity of the system, which has exposed them to a high financial risk [31]. The internal migrants without social health insurance usually have lower levels of use of both medical services and preventive care compared with their counterparts with social health insurance [8,17,32,33,34,35,36,37]. Some internal migrants were covered by the BMIUE, which enabled them to enjoy a higher level of entitlements and use more healthcare services than those covered by the BMIUR and RNCMS [36,37,38]. The use of healthcare services by internal migrants is further complicated by the location of insurance funds. Empirical evidence shows that the internal migrants who have enrolled with a social health insurance program outside of their residential location are likely to abandon visits to physicians when needed because of difficulties in accessing the insurance funds [39,40].

Recently, the Chinese government developed a series of policies to address the inequality concerns. These include initiatives to enable internal migrants to obtain better access to local health services [7,11]. The BMIUR and RNCMS, for example, have merged in many local governmental catchments although the BMIUE remains separated [14,41]. Meanwhile, insurance funds are encouraged to improve efficiency in fund management and to allow transfer of insurance funds for internal migrants [42]. Internal migrants can choose to enroll in a local social health insurance program outside of their Hukou location [43,44,45,46]. Despite high expectations on these new arrangements [14,39,47], early evaluations demonstrated slow progress. Many internal migrants found it difficult to take advantage of the new arrangements [48,49]. It was estimated that in 2017 more than 66% of internal migrants stayed with their social health insurance schemes in their Hukou location [12,31,50,51]. Policy unawareness, fragmentation of insurance policies, high financial costs of fund change, perceived low needs, job insecurity, and high population mobility may have all contributed to the low enrolments in local health insurance [3,52]. As a result, on-the-spot settlements of medical bills emerged as a compromised solution to the mismatch between fund location and provision of healthcare services [52,53,54]. The on-the-spot settlements of medical bills started in 2014 first within the geographic catchment of a municipality, followed by a provincial-wide arrangement in 2015. In 2016, it became a nationwide arrangement [47,55]. The preliminary evidence showed that the on-the-spot settlements system has indeed stimulated use of medical services by internal immigrants [47,55]. However, its effect remains unclear [2,14,39,47]. In addition, there is paucity in the literature documenting how the insurance arrangements influence health seeking behaviors of the majority of internal migrants, despite some studies into the elderly populations of internal migrants [39,40,47]. Most existing studies reported a higher level of use of medical services in the elderly migrants who enrolled with a local health insurance program than those who did not [39,40,47].

This study aimed to estimate the effect of the on-the-spot medical bill settlement system on the access to both medical services and preventive care by the internal migrants through comparing those with and without local health insurance coverage. The study advances our understanding of the healthcare seeking behaviors of internal migrants by unpacking the components of health services into illness treatments and preventive care. Theoretically, inequalities in preventive care are likely to cause more serious damages to population health than those in illness treatments [56]. However, the impacts of health insurance arrangements on the two components of healthcare services are likely to vary as governmental grants for public health can be drawn to cover preventive care. The study also expands our views on inequalities to those within the internal migrant populations, which can offer some insight into the potential strategies for reducing inequalities. We hypothesized that the effects of the on-the-spot medical bill settlements vary between the rural-to-urban and the urban-to-urban migrants. Unlike previous studies that restricted participants to those aged 60 years and older prior to the nation-wide settlement arrangements [39,40,47,57], our study extended the sample to all of the adult participants and was conducted one year after the national on-the-spot medical bill settlement system was implemented. It was estimated that more than 96% of internal migrants are younger than 60 years. They usually have quite different healthcare needs and service-seeking behaviors compared to their older counterparts [58]. To the best of our knowledge, this study is the first of its kind in China, estimating the effect size of insurance fund location on use of health services in a large nationally representative sample after the implementation of the nationwide on-the-spot medical bill settlement system.

## 2. Materials and Methods

### 2.1. Study Design and Data Source

Data were obtained from the 2017 China Migrants Dynamic Survey (CMDS). The CMDS is a large nationwide cross-sectional questionnaire survey on internal migrants conducted by the National Health Commission of China every year since 2009 [1,2]. The survey contains questions about the demographic and socioeconomic characteristics of the respondents and their family members, as well as their health status and use of health services. Eligible participants of this study included those who were 15 years or older, resided in a residential address outside of their Hukou location (urban district or rural county) for more than one month, and completed the questionnaire on behalf of their households [12,59].

#### 2.1.1. Setting and Sampling

The 2017 CMDS survey sample was drawn based on the 2016 China Migrant Population Information System. A multistage stratified probability proportional-to-size (PPS) cluster sampling strategy was adopted to select participants [8,59]. At the first stage, 3150 urban sub-districts and rural townships were selected using the PPS method from 1290 counties/districts of 351 prefectures in 32 provinces. This was followed by a PPS selection of 8500 residential communities/villages from the participating sub-districts/townships. At the third stage, 20 migrant households were randomly sampled from each participating community/village. One respondent (≥15 years of age) from each household was invited to complete the questionnaire. A total of 169,989 completed questionnaires were returned with a response rate of 99% [1,59]. In this study, we excluded those (13,918, 8.2%) without social health insurance and those (11,115, 6.5%) with more than one social health insurance programs. This resulted in a final sample of 144,956 for data analyses, representing 84.4% of those being surveyed.

#### 2.1.2. Data Collection

The questionnaire was administered through face-to-face interviews using the Computer Assisted Personal Interviewing (CAPI) technique. All of the interviewers were rigorously selected and trained to ensure that they followed the standard protocol [59]. The eligibility of the interviewers was assessed through an examination that demonstrated their proper understanding of the questionnaire and ability to meet the data collection standards developed by the National Health Commission of China. The interviews were conducted in the households of the respondents. Oral informed consent was obtained from each participant prior to the interview. The identified participants who were not accessible were allowed to be replaced by those with similar characteristics in terms of age, gender, location and residential status, preferably from the database recorded in the 2016 China Migrant Population Information System.

A rigorous data verification process was established through the coordination of the National Health Commission of China. The CAPI system identified logical errors that fed back to the interviewers. The completed questionnaires were then checked by a quality assurance officer at each sub-district/township or district/county. The survey supervisors at the provincial or national level performed additional audits on the returned questionnaires through random telephone interviews or site visits. Further details about the survey can be found in the “Handbook of China Migrants Dynamic Survey 2017” [59].

### 2.2. Data Analysis

The Andersen’s behavioral model, a conceptual framework illustrating the determinants of health services usage, was adopted in this study to guide the construction of statistical models and the selection of indicators/variables [60,61]. This study examined the health behaviors of internal migrants (dependent variables) in terms of medical services for illness treatment and preventive care for maintaining health in line with the defined policy targets of the Chinese government in health development and those used in previous studies [62,63,64,65]. According to the Andersen model [60,61], health behaviors are determined by predisposing (e.g., age and sex), enabling (e.g., income and insurance) and needs (e.g., health and illness conditions) factors. The CMDS data were mapped into the three categories of determinants and served as independent variables.

#### 2.2.1. Dependent Variables

In this study, two indictors were calculated to measure use of health services in line with those used in previous studies [8,17,32,33,34,35,36,37].

(1) Registration for essential public health services—this is a proxy indicator measuring access to a package of essential public health services. Respondents were asked whether they had a health record established in the local community health center (1 = yes, 0 = no) [59]. A health record recorded the social and health status of the community resident. It enabled a community health center to deliver essential public health services (e.g., screening and management of chronic conditions) to those who had been recorded. The local governments allocated per capita funding to support a range of population based health care services, targeting the priority areas identified through the personal health records [17].

(2) Visits to physicians when needed as a migrant—this indicator reflects “realized access” to medical care [65]. The data analysis was restricted to the respondents who reported illness whilst living in the immigrated location (n = 57,664). They were asked “What did you do when you felt ill or injured in the last time?”. A response of “visiting a physician” (regardless of which facilities) was coded as “1”. For those who sought self-medication or did not seek medical attention, a code of “0” was assigned [59].

#### 2.2.2. Independent and Control Variables

The effects of health insurance arrangements on the use of health services were the major focus of this study. The location of insurance funds was selected to measure health insurance arrangements and served as the independent variable. Respondents were asked whether they enrolled in a social health fund at their residential location (1 = yes; 0 = no).

The control variables were selected according to the Andersen model [60,61], which were used for the purpose of adjustment of confounding effects. In this study, gender, age and marital status fell into the category of predisposing factors, while educational attainment, employment, household income, and type of social health insurance were considered as enabling factors. Health needs were measured using three indicators: self-rating health, two-week morbidity and chronic conditions. Respondents were asked to report their gender (male vs female), age (15–24, 25–34, 35–44, 45–54, 55+ years), marital status (never married/single, married, divorced, widowed), educational attainment (illiterate, primary school, junior middle school, senior middle school, university/college), employment (employed, unemployed), household income, and type of insurance funds (BMIUE, BMIUR/RNCMS, and others). They were also asked to rate their overall health (good, general, poor), and to report experiences of acute illness over a two-week period (no or yes) and chronic conditions (hypertension and diabetes) diagnosed by a doctor (no or yes). The data about household income were transformed into a ranking within respective province (<percentile 20, percentile 20–39, percentile 40–59, percentile 60–79, and ≥percentile 80) for data analyses.

Significant regional variations in social and health development exist in China. Such contextual characteristics could play a significant role in shaping the use of health services. Therefore, contextual characteristics were included as another layer of control variables (enabling factor) in this study. Immigrated destination (counties/districts) served as an indicator reflecting the contextual differences of the study participants. Each county/district would have its special socioeconomic environment and insurance policies, including rules about fund transfer and portability of insurance entitlements. However, further inclusion of these more specific variables in data analyses would simply overlap with the county/district variable.

#### 2.2.3. Statistical Analysis

Multilevel modelling has been widely used in analyzing data with a cluster structure [56,66]. In this study, two-level logistic regression models were established to compare the use of health services between the internal migrants with and without local health insurance, after adjustment for variations in the control variables at the individual level (fixed effects) and the county/district level (random effect).

We constructed an empty model first with only a random intercept to determine whether the contextual dimension was appropriate to be treated as the second level. The intraclass correlation coefficients (ICCs) of the empty models showed statistical significance, indicating that a two-level modeling was appropriate for this study [56,67]: 0.45 for having a health record and 0.10 for the use of medical services at the county/district level. The two-level logistic regression models were then established as the following:

(1) $$\log it(Y_{ij})=(\beta_{0}+\beta_{place}X_{place\_ij})+\sum_{h=1}^{n}\beta_{control\_h}X_{control\_hij}+(u_{0j}+u_{place\_j}X_{place\_ij})+\varepsilon_{0ij}$$

This model estimates the main effect of insurance fund location, where *Y_ij_* represents the use of health services at an individual level (*i* refers to each respondent, *j* refers to each county/district). *X_place_ij_* indicates the location of health insurance fund enrolled by respondent *i* in county/district *j*. *X_control_hij_* represents the control variables entered into the models, including gender, age, educational attainment, marital status, employment, ranking of household income, type of health insurance, self-reported health, two-week morbidity, and chronic conditions. *β_place_* and *β_control_h_* represent the fixed effects of the location of health insurance funds and control variables on the use of health services at the individual level (level 1), respectively. *u_place_j_* indicates the random effect of *X_place_ij_* on the use of health services at the contextual level (level 2). *β*_0_ is the fixed intercept. *u*_0*_j*_ indicates the random intercept. *ε*_0*ij*_ is the random error at level 1.

We then added the interaction effects between location and type of health insurance funds into the models in order to better understand the effects of cross-county/district insurance arrangements. We also performed sub-group analyses, comparing the effects of insurance arrangements on the rural-to-urban and urban-to-urban migrants.

All analyses were performed using STATA version 16.0 (SE) for Windows (StataCorp LLC, College Station, TX, USA). The pweight method based on the sampling design was used to weigh the cases and the robust method was use to estimate variance–covariance matrix (VCE) corresponding to the parameter estimates [68]. The effects of health insurance fund location on the use of health services were presented using adjusted odds ratio (AOR) and average marginal effects (AME) [39]. A higher AOR or AME indicates a higher effect. The statistical significance level was set at 0.05.

## 3. Results

### 3.1. Characteristics of Respondents

Slightly more than half of the respondents (51.65%) were male. About 6% of the respondents were older than 55 years; 17% had a university degree. The majority (83.26%) were married at the time of the survey. Although most (82.18%) of the respondents had a job, only 18.35% were enrolled with the BMIUE. Rural-to-urban migrants accounted for about 80% of the respondents (Table 1).

Less than one-third (30.28%) of the respondents had a health record. More than half (50.77%) visited a physician when they felt ill or were injured. About 24% of the respondents enrolled in a social health insurance scheme locally, wherever they resided. Those who were female (*p* < 0.05), young, healthy, received higher education, single, employed, had higher income, enrolled with the BMIUE, and immigrated from urban to urban (*p* < 0.001) were more likely to enroll in a health insurance scheme locally (Table 1).

### 3.2. Having a Health Record

The respondents with local health insurance coverage were more likely to have a health record than those without local health insurance coverage (35.31% vs. 28.61%, *p* < 0.001). The odds of having a health record in the respondents with local health insurance coverage were 1.47 times (AOR = 1.47, 95% CI = 1.30–1.65) of those without local health insurance coverage: 6.1 percentage points higher (AME = 6.1%, 95% CI: 4.3–7.8%) after controlling for variations in other variables. The effect sizes were similar between the rural-to-urban (AME = 6.8%, 95% CI: 4.9–8.6%) and the urban-to-urban (AME = 6.8%, 95% CI: 3.2–10.4%) migrants (Table 2).

The contextual variable “county/district” contributed to 44% of the total variance of the dependent variable (having heath records): 45% for the rural-to-urban migrants and 37% for the urban-to-urban migrants. The regression models also showed that female migrants were more likely to have a health record than male migrants (AOR = 1.21, *p* < 0.001). Those who were married (AOR = 1.32, *p* < 0.001), had higher levels of education (AOR = 1.41–2.08, *p* < 0.001), and had higher household income (AOR = 1.02–1.11, *p* < 0.05) were more likely to have a health record than others. The respondents who reported worse health were less likely to have a health record than those reporting better health (AOR = 0.73–0.80, *p* < 0.05). Chronic morbidity was associated with higher odds of having health records (AOR = 1.30, *p* < 0.001). These effects were consistent between the rural-to-urban and the urban-to-urban migrants (Table 2).

Although the type of health insurance had no significant association with the establishment of health records (*p* > 0.05), the interaction effect between location and type of health insurance on the establishment of health records was statistically significant (Supplementary File Tables S1 and S2). The location effect was lower on the establishment of health records in those covered by the BMIUE (AOR = 0.71, *p =* 0.01, Table S1) in comparison with those covered by the merged insurance BMIUR/RNCMS.

### 3.3. Use of Medical Services

The respondents with local health insurance coverage were more likely to visit physicians when needed (AOR = 1.18, 95% CI = 1.06–1.30): 3.5 percentage points higher than those without local health insurance coverage after controlling for variations in other variables (AME = 3.5%, 95% CI: 1.3–5.8%). However, such an association was only statistically significant in the rural-to-urban migrants (AME = 4.8%, 95% CI: 1.8–7.7%) (Table 3).

The contextual variable “county/district” contributed to 10% of the total variance of the dependent variable (visits to physicians): 10% for the rural-to-urban migrants and 11% for the urban-to-urban migrants (Table 3). The regression models also revealed that female migrants were more likely to visit a physician when they felt ill or were injured compared with their male counterparts (AOR = 1.08, *p* = 0.01). Those who were covered by the BMIUE were less likely to visit a medical doctor than those covered by the BMIUR/RNCMS (AOR = 0.85, *p* < 0.001). The respondents experiencing health problems, such as those who worse self-rated health (AOR = 1.14–1.80, *p* < 0.001), reported two-week morbidity (AOR = 1.21, *p* < 0.001), and had chronic morbidity (AOR = 1.33, *p* < 0.001) were more likely to visit physicians when needed. These effects were consistent between the rural-to-urban and the urban-to-urban migrants, despite a lack of statistical significance in the gender effect (AOR = 1.06, *p* = 0.10) for the rural-to-urban migrants and the effect of type of insurance (AOR = 0.95, *p* = 0.62) for the urban-to-urban migrants (Table 3).

The interaction effect between location and type of health insurance on visits to physicians was statistically significant (Supplementary File Tables S3 and S4). The location effect was higher on visits to physicians in those covered by the BMIUE in comparison with those covered by the merged insurance BMIUR/RNCMS, but only statistically significant in the rural-to-urban migrants (AOR = 1.54, *p* = 0.01, Table S3).

## 4. Discussion

This study provides some new insight into the outcomes of the new health insurance arrangements in China that aim at improving health services for internal migrants. The results show that there still exist gaps in the use of health services, both in medical treatments and in preventive care, between the internal migrants with and without local health insurance coverage one year after the implementation of the nationwide on-the-spot medical bill settlement system. The internal migrants covered by a health insurance program outside of their residential location tend to use less healthcare services. Such a disparity varies by the type of insurance and between the rural-to-urban and the urban-to-urban migrants. The contextual variable “county/district” contributes to 44% of the variance of the establishment of health records and 10% of the variance of visiting to physicians when needed, although household income was not identified as a significant predictor of visits to physicians.

The inequality in use of health services deserves further policy attention. Our data show that the overall level of health services used by the internal migrants is low: only about half of the internal migrants visited a medical doctor when needed, compared with a national average of 84.5% (in 2013) [69]. The low coverage (31.28%) of health records in this population is particularly concerning because it is unlikely that these migrants are able to obtain access to the essential public health services (such as case management of chronic conditions) outside of one’s residential location [69,70,71]. On average, almost 70% of the entire population in China had been covered by a health record in 2013 [69]. The inequality in health services may further exacerbate the existing gaps in health and health risks between internal migrants and the locals. This has been considered as a serious risk amid the recent outbreak of COVID-19. Rural-to-urban migrants are usually exposed to high risks of infectious diseases [72]. The hesitation of internal migrants in seeking local medical attention could seriously jeopardize the public health efforts to contain the spread of infectious diseases [72,73]. To address this problem, the Chinese government has made testing and hospital services free of charge to everybody during the COVID-19 crisis [74] through subsidizing the insurance funds [75]. However, it is imperative to prepare for future challenges by tackling the fundamental shortfalls of the social health insurance arrangements.

The on-the-spot settlement system of medical bills appears to have not yet achieved its full potential. This study shows that the inequality in use of health services between the migrants with and without local insurance coverage still existed one year after the introduction of the system. The internal migrants without local health insurance coverage used significantly less healthcare services. This may be caused by the existence of thousands of social health insurance funds and a lack of coordination and coherent policies in China [27]. Although a nationwide network has been established to facilitate on-the-spot settlements of medical bills across regions, great barriers exist for the internal migrants to take advantage of the new arrangements. Some researchers call for expansion of the limited number of hospitals assigned by the national health insurance authority for the on-the-spot medical bill settlements [48,76]. The restriction of the on-the-spot medical bill settlements to hospital admissions for acute illness is also attracting increasing criticisms. For services other than acute hospital admissions, patients have to pay out of pocket before asking for a proportion of reimbursements from their enrolled fund. In addition, many internal migrants are not even aware of the relevant policies and procedures, let alone how to navigate through the complex processes [40]. However, the gaps appear to have decreased in comparison with the findings reported in a previous study [39]. We found a gap of 3.5 percentage points (adjusted for variations in the control variables) in use of medical services between the migrants with and without local insurance coverage, much smaller than the 7.4 percentage point gap reported prior to the introduction of the on-the-spot bill settlement system.

It is worth noting that the association between local health insurance enrolment and use of health services is stronger in the rural-to-urban migrants than in the urban-to-urban migrants. The gap in visiting physicians when needed between the urban-to-urban migrants with and without local insurance coverage has virtually closed one year after the introduction of the system. China has a long history of dual welfare systems. Urban residents are entitled to higher levels of welfare and income compared to their rural counterparts. This makes rural-to-urban migrants particularly vulnerable to insurance arrangements: they are more likely to abandon health services due to financial barriers arising from the failure of the on-the-spot medical bill settlements [38]. Although the BMIUE programs are often more generous and can cover medical services outside of the fund location [26,41,42,77], very few rural-to-urban migrants are allowed to enroll with the BMIUE. This may jeopardize the potential effect of the local insurance enrolment policy on use of health services [38,77,78].

In theory, the merging of the BMIUR and the RNCMS may be able to mitigate some of the consequences of the mismatch between insurance fund location and provision of health services. This may be true for medical services for illness treatments. However, this study shows that the location effect was lower on the establishment of health records in those covered by the BMIUE in comparison with those covered by the merged insurance BMIUR/RNCMS. Clearly, preventive care has not been targeted by the merging of the two insurance programs. Unlike medical services, access to health records is less likely to be influenced by insurance arrangements directly. While the BMIUE enrollees may be able to enjoy public health services offered by their employers, the only way for the BMIUR and RNCMS enrollees to receive public health services is through their local primary care facilities. Unfortunately, the rural-to-urban migrants who hold a rural household registration may not be seen as urban “locals” even if the BMIUR and RNCMS programs are merged.

The findings of this study have some policy implications for China’s health insurance development. Neither local enrollment of social health insurance nor on-the-spot settlements of medical bills would offer a perfect solution to the inequality problems in use of health services by internal migrants in China. Low coverage (<25%) of local health insurance in internal migrants is evident, defying the policy encouragement of the Chinese government [44,45,46]. The existence of numerous health insurance funds has created a significant challenge to the portability arrangement of insurance funds, in particular between urban and rural. However, it is important to note that the migrants who endorse themselves as locals (as evidenced by joining local insurance) are more likely to have a health record and consequently receive local public health services and medical services despite the absence of a direct link between insurance arrangements and health records. This study revealed a greater gap in registration with public health services compared with that in medical services between those with and without local insurance coverage. Fund location has a greater association with use of health services in the rural-to-urban migrants compared with that in the urban-to-urban migrants.

In recent years, there has been an increasing call for further consolidation of the existing health insurance funds both vertically and horizontally at a higher administrative level [26,41]. Although consolidation of health insurance funds may offer a fundamental solution to the problems in health services experienced by internal immigrants [41], some transitional strategies need to be developed urgently in line with their diverse needs [25]. For example, local enrollments in the BMIUE can be made compulsory for those who have an employment contract, while others can be encouraged to enroll with local BMIUR. Local enrollments can also be encouraged through easier and fairer fund transfer arrangements. Meanwhile, a cross-regional fund can be established to simplify on-the-spot settlements of medical bills and extend its coverage beyond hospital admissions for acute illness [47]. Such arrangements are particularly important for the rural-to-urban migrants who change jobs and residential locations frequently and find it hard to enroll in an insurance scheme locally. However, this will require a coordinated risk sharing mechanism across funds with varied levels of premium and entitlement policies [52]. In addition, it is important for the public to participate in policy dialogues and contribute to the development of more appropriate insurance arrangements tailored to the diverse needs of the internal migrants.

This study provides a comprehensive assessment on inequalities in healthcare services within the internal migrant populations and in comparison with the average of the entire population in China. It highlights the importance of including preventive care in inequality studies. Healthcare seeking behaviors can be shaped by many factors, including the inherent link between preventive care and illness treatments. A better alignment between health insurance policies and public health activities should be advocated. This will require a whole-of-government approach to welfare system development. 

There are several limitations in this study. Due to unavailability of data, only self-reported health status was included in risk-adjustment of the findings. We were not able to decompose the outcome indicators into more specific health services. Medical expenses were not analyzed either. The study adopted a cross-sectional design, no causal relationships should be assumed. We cannot rule out the possible influence of selection bias on the effect of local health insurance: those who intend to use more services may be more likely to enroll with a local insurance scheme. Further studies are needed to investigate how on-the-spot settlements of medical bills are implemented through coordination across multiple insurance funds.

## 5. Conclusions

In conclusion, although on-the-spot settlements of medical bills have been encouraged by the Chinese government, inequalities in the use of both medical and public health services still exist among internal migrants with and without local social health insurance coverage. Higher levels of use of medical and public health services are associated with local health insurance coverage. The impact of local insurance enrollments is greater in the rural-to-urban migrants compared with the urban-to-urban migrants, especially for the use of medical services. Meanwhile, the effects of local insurance enrollments also vary with the type of insurance. The low coverage of health records in internal migrants is particularly concerning because it may further exacerbate the existing gaps in health and health risks between internal migrants and the local population. Further studies are needed to investigate how on-the-spot settlements of medical bills are implemented through coordination across multiple insurance funds and how public health services can be improved for internal migrants.

## Figures and Tables

**Table 1:** Characteristics of respondents with and without local social health insurance.

Variable	Description	Sample Size		Number (%) of Respondents		Number (%) of Respondents		χ^2^ Value	*p* Value
		n	%	without Local Insurance		with Local Insurance			
Gender								7.61	0.01
	Male	74,864	51.65	57,319	(51.85)	17,545	(51.00)		
	Female	70,092	48.35	53,233	(48.15)	16,859	(49.00)		
Age (Years)								1400	<0.001
	15–24	17,952	12.38	14,103	(12.76)	3849	(11.19)		
	25–34	54,823	37.82	39,180	(35.44)	15,643	(45.47)		
	35–44	39,393	27.18	30,247	(27.36)	9146	(26.58)		
	45–54	24,433	16.86	20,070	(18.15)	4363	(12.68)		
	55+	8355	5.76	6952	(6.29)	1403	(4.08)		
Marital status								369.67	<0.001
	Never married/Single	20,340	14.03	14,458	(13.08)	5882	(17.10)		
	Married	120,687	83.26	93,030	(84.15)	27,657	(80.39)		
	Divorced	2591	1.79	1969	(1.78)	622	(1.81)		
	Widowed	1338	0.92	1095	(0.99)	243	(0.71)		
Educational attainment								18,000	<0.001
	Illiterate	3994	2.76	3500	(3.17)	494	(1.44)		
	Primary school	21,460	14.8	18,601	(16.83)	2859	(8.31)		
	Junior middle school	64,450	44.46	54,660	(49.44)	9790	(28.46)		
	Senior middle school	31,178	21.51	23,268	(21.05)	7910	(22.99)		
	University/college	23,874	16.47	10,523	(9.52)	13,351	(38.81)		
Employment								850.73	<0.001
	Unemployed	25,837	17.82	21,513	(19.46)	4324	(12.57)		
	Employed	119,119	82.18	89,039	(80.54)	30,080	(87.43)		
Household income ranking								3000	<0.001
	Lowest (<percentile 20)	33,249	22.94	27,658	(25.02)	5591	(16.25)		
	Lower (percentile 20–39)	30,504	21.04	24,548	(22.20)	5956	(17.31)		
	Middle (percentile 40–59)	27,913	19.26	21,406	(19.36)	6507	(18.91)		
	Higher (percentile 60–79)	27,771	19.16	20,239	(18.31)	7532	(21.89)		
	Highest (≥percentile 80)	25,519	17.6	16701	(15.11)	8818	(25.63)		
Type of social health insurance								69,000	<0.001
	BMIUR/RNCMS *	118,123	81.49	106,546	(96.38)	11,577	(33.65)		
	BMIUE	26,594	18.35	3874	(3.50)	22,720	(66.04)		
	Others	239	0.16	132	(0.12)	107	(0.31)		
Self-rating of health								136.8	<0.001
	Good	118,780	81.94	89,901	(81.32)	28,879	(83.94)		
	General	22,123	15.26	17,357	(15.70)	4766	(13.85)		
	Poor	4053	2.8	3294	(2.98)	759	(2.21)		
Two-week morbidity								0.66	0.42
	No	135,728	93.63	103,482	(93.60)	32,246	(93.73)		
	Yes	9228	6.37	7070	(6.40)	2158	(6.27)		
Chronic morbidity								59.71	<0.001
	No	136,874	94.42	104,101	(94.16)	32,773	(95.26)		
	Yes	8082	5.58	6451	(5.84)	1631	(4.74)		
Type of migration								8700	<0.001
	Rural to urban	114,153	78.75	93,233	(84.33)	20,920	(60.81)		
	Urban to urban	30,803	21.25	17,319	(15.67)	13,484	(39.19)		
Having a health record								524.63	<0.001
	No	91,925	69.72	70,635	(71.39)	21,290	(64.69)		
	Yes	39,933	30.28	28,312	(28.61)	11,621	(35.31)		
Visits to physicians								1.83	0.18
	No	34,634	49.23	25,649	(49.08)	8985	(49.66)		
	Yes	35,722	50.77	26,614	(50.92)	9108	(50.34)		
**Total**		144,956	100	110,552	(100.00)	34,404	(100.00)		

Note: * BMIUR–Basic Medical Insurance for Urban Residents; RNCMS–Rural New Cooperative Medical Scheme; BMIUE–Basic Medical Insurance for Urban Employees.

**Table 2:** Factors associated with the establishment of personal health records: results of two-level logistic regression models.

Variables		Rural to Urban Respondents					Urban to Urban Respondents					All Respondents				
		AOR/ICC ^†^	SE	*p* Value	95%CI		AOR/ICC	SE	*p* Value	95%CI		AOR/ICC	SE	*p* Value	95%CI	
Fixed effects (Level 1)																
Enrolment with a local social health insurance program	No (reference)															
	Yes *	1.56	0.10	<0.001	1.38	1.77	1.46	0.17	<0.001	1.16	1.83	1.47	0.09	<0.001	1.30	1.65
Gender	Male (reference)															
	Female	1.21	0.04	<0.001	1.13	1.29	1.19	0.07	<0.001	1.06	1.33	1.21	0.03	<0.001	1.14	1.28
Age (Years)	15–24 (reference)															
	25–34	1.00	0.07	0.95	0.87	1.15	1.10	0.12	0.38	0.89	1.36	1.01	0.07	0.85	0.88	1.16
	35–44	1.04	0.08	0.62	0.90	1.20	1.01	0.12	0.92	0.80	1.27	1.02	0.08	0.79	0.88	1.18
	45–54	0.93	0.07	0.39	0.80	1.09	1.02	0.15	0.89	0.76	1.36	0.95	0.07	0.45	0.82	1.09
	55+	1.09	0.15	0.51	0.84	1.43	1.31	0.19	0.07	0.98	1.75	1.20	0.14	0.13	0.95	1.51
Marital status	Never married/Single (reference)															
	Married	1.31	0.09	<0.001	1.14	1.50	1.31	0.14	0.01	1.06	1.62	1.32	0.10	<0.001	1.14	1.53
	Divorced	1.19	0.15	0.18	0.92	1.54	1.38	0.24	0.07	0.98	1.94	1.26	0.15	0.05	1.00	1.58
	Widowed	1.12	0.22	0.55	0.77	1.64	1.22	0.42	0.57	0.61	2.41	1.12	0.20	0.52	0.80	1.58
Educational attainment	Illiterate (reference)															
	Primary school	1.34	0.14	0.01	1.09	1.64	1.58	0.58	0.22	0.77	3.25	1.41	0.14	<0.001	1.16	1.71
	Junior middle school	1.55	0.16	<0.001	1.27	1.90	2.02	0.73	0.05	1.00	4.09	1.66	0.16	<0.001	1.37	2.01
	Senior middle school	1.84	0.22	<0.001	1.45	2.33	2.15	0.79	0.04	1.05	4.42	1.94	0.22	<0.001	1.56	2.42
	University/college	1.97	0.24	<0.001	1.54	2.50	2.28	0.88	0.03	1.07	4.85	2.08	0.23	<0.001	1.67	2.57
Employment	Unemployed (reference)															
	Employed	0.94	0.04	0.11	0.86	1.01	0.98	0.08	0.78	0.83	1.15	0.93	0.03	0.06	0.87	1.00
Household income ranking	Lowest (<percentile 20, reference)															
	Lower (percentile 20–39.9)	1.13	0.05	0.01	1.03	1.24	1.02	0.07	0.80	0.89	1.17	1.11	0.05	0.01	1.02	1.20
	Middle (percentile 40–59.9)	1.12	0.05	0.01	1.03	1.23	1.03	0.10	0.79	0.85	1.25	1.10	0.05	0.02	1.01	1.20
	Higher (percentile 60–79.9)	1.01	0.05	0.76	0.93	1.11	1.02	0.10	0.88	0.84	1.23	1.02	0.04	0.61	0.94	1.11
	Highest (≥percentile 80)	1.11	0.07	0.10	0.98	1.25	0.93	0.10	0.52	0.75	1.16	1.06	0.06	0.31	0.95	1.17
Type of social health insurance	BMIUR/RNCMS (reference)															
	BMIUE	1.05	0.10	0.59	0.88	1.26	1.03	0.14	0.82	0.79	1.34	1.09	0.10	0.34	0.91	1.30
	Others	2.09	1.58	0.33	0.48	9.16	1.27	0.45	0.50	0.63	2.54	1.43	0.48	0.29	0.74	2.76
Self-rating of health	Good (reference)															
	General	0.75	0.04	<0.001	0.67	0.84	0.66	0.04	<0.001	0.58	0.75	0.73	0.03	<0.001	0.67	0.80
	Poor	0.83	0.08	0.05	0.69	1.00	0.74	0.12	0.08	0.53	1.03	0.80	0.06	0.01	0.69	0.93
Two-week morbidity	No (reference)															
	Yes	1.01	0.09	0.92	0.85	1.20	1.11	0.14	0.40	0.87	1.41	1.04	0.08	0.63	0.89	1.21
Chronic morbidity	No (reference)															
	Yes	1.24	0.07	<0.001	1.11	1.38	1.44	0.17	<0.001	1.13	1.82	1.30	0.06	<0.001	1.18	1.43
Radom effects (Level 2)																
	Variance (enrolment with local social health insurance)	0.57	0.08		0.44	0.75	0.70	0.15		0.46	1.07	0.58	0.11		0.39	0.85
	Variance (intercept)	2.74	0.18		2.42	3.11	1.92	0.17		1.62	2.27	2.56	0.18		2.22	2.95
ICC																
	Empty model	0.45	0.02		0.42	0.48	0.36	0.02		0.32	0.40	0.45	0.01		0.42	0.48
	Full model	0.45	0.02		0.42	0.49	0.37	0.02		0.33	0.41	0.44	0.02		0.40	0.47
Wald Chi Square test																
	Chi-Square	372.43					184.3					422.56				
	*p* value	<0.001					<0.001					<0.001				

Note: * Average marginal effects (AME) of location of social health insurance funds: 6.8% (4.9%, 8.6%) for rural-to-urban respondents; 6.8% (3.2%, 10.4%) for urban-to-urban respondents; 6.1% (4.3%, 7.8%) for all respondents. ^†^ ICC: intraclass correlation coefficient; AOR: adjusted odds ratio; SE: standard error; CI: confidence interval.

**Table 3:** Factors associated with visits to local physicians when needed: results of two-level logistic regression models.

Variables		Rural to Urban Respondents					Urban to Urban Respondents					All Respondents				
		AOR/ICC ^†^	SE	*p* Value	95%CI		AOR/ICC	SE	*p* Value	95%CI		AOR/ICC	SE	*p* Value	95%CI	
Fixed effects (Level 1)																
Enrolment with a local social health insurance program	No (reference)															
	Yes *	1.24	0.08	<0.001	1.09	1.42	1.09	0.09	0.32	0.92	1.28	1.18	0.06	<0.001	1.06	1.30
Gender	Male (reference)															
	Female	1.06	0.04	0.10	0.99	1.13	1.15	0.07	0.03	1.02	1.30	1.08	0.03	0.01	1.02	1.14
Age (Years)	15–24 (reference)															
	25–34	1.09	0.08	0.21	0.95	1.25	0.90	0.13	0.46	0.68	1.19	1.05	0.06	0.42	0.93	1.18
	35–44	1.05	0.08	0.47	0.91	1.22	0.82	0.13	0.21	0.61	1.12	1.00	0.07	0.98	0.87	1.14
	45–54	0.91	0.07	0.24	0.79	1.06	0.84	0.13	0.28	0.62	1.15	0.89	0.06	0.10	0.78	1.02
	55+	1.02	0.11	0.88	0.82	1.27	0.85	0.17	0.42	0.58	1.26	0.97	0.09	0.75	0.82	1.16
Marital status	Never married/Single (reference)															
	Married	1.02	0.08	0.75	0.89	1.18	1.08	0.13	0.55	0.85	1.37	1.03	0.08	0.73	0.89	1.18
	Divorced	1.03	0.14	0.83	0.78	1.36	1.13	0.28	0.63	0.70	1.83	1.04	0.13	0.78	0.81	1.32
	Widowed	0.90	0.19	0.60	0.60	1.34	1.47	0.41	0.17	0.85	2.53	0.99	0.18	0.98	0.70	1.41
Educational attainment	Illiterate (reference)															
	Primary school	1.05	0.10	0.61	0.87	1.27	0.64	0.19	0.13	0.36	1.14	1.01	0.10	0.94	0.84	1.21
	Junior middle school	1.14	0.11	0.17	0.94	1.38	0.66	0.20	0.16	0.37	1.18	1.09	0.10	0.32	0.92	1.29
	Senior middle school	1.09	0.15	0.52	0.84	1.43	0.72	0.21	0.27	0.41	1.29	1.06	0.12	0.61	0.85	1.31
	University/college	1.12	0.13	0.37	0.88	1.41	0.74	0.23	0.32	0.40	1.34	1.07	0.10	0.46	0.89	1.29
Employment	Unemployed (reference)															
	Employed	0.89	0.04	0.02	0.81	0.98	1.13	0.12	0.24	0.92	1.40	0.94	0.04	0.13	0.86	1.02
Household income ranking	Lowest (<percentile 20, reference)															
	Lower (percentile 20–39.9)	1.02	0.05	0.74	0.93	1.11	1.14	0.13	0.24	0.92	1.42	1.03	0.05	0.49	0.95	1.12
	Middle (percentile 40–59.9)	1.00	0.06	0.95	0.90	1.12	1.15	0.14	0.24	0.91	1.46	1.03	0.05	0.63	0.93	1.14
	Higher (percentile 60–79.9)	1.02	0.05	0.65	0.93	1.13	1.03	0.11	0.78	0.84	1.26	1.01	0.05	0.82	0.92	1.11
	Highest (≥percentile 80)	1.07	0.08	0.42	0.92	1.24	0.96	0.12	0.77	0.76	1.23	1.01	0.07	0.84	0.88	1.17
Type of social health insurance	BMIUR/RNCMS (reference)															
	BMIUE	0.83	0.07	0.02	0.71	0.97	0.95	0.09	0.62	0.80	1.14	0.85	0.05	<0.001	0.76	0.95
	Others	6.43	4.49	0.01	1.63	25.29	1.06	0.24	0.81	0.68	1.64	1.19	0.23	0.36	0.82	1.75
Self-rating of health	Good (reference)															
	General	1.15	0.06	0.01	1.04	1.27	1.16	0.10	0.10	0.97	1.39	1.14	0.05	<0.001	1.05	1.25
	Poor	1.81	0.18	<0.001	1.49	2.20	1.87	0.45	0.01	1.16	3.01	1.80	0.19	<0.001	1.47	2.20
Two-week morbidity	No (reference)															
	Yes	1.21	0.06	<0.001	1.10	1.34	1.20	0.11	0.05	1.00	1.45	1.21	0.06	<0.001	1.10	1.32
Chronic morbidity	No (reference)															
	Yes	1.30	0.10	<0.001	1.12	1.51	1.39	0.18	0.01	1.08	1.80	1.33	0.09	<0.001	1.16	1.52
Radom effects (Level 2)																
	Variance (enrolment with local social health insurance)	0.36	0.07		0.25	0.52	0.21	0.07		0.11	0.41	0.26	0.04		0.19	0.36
	Variance (intercept)	0.37	0.04		0.31	0.45	0.39	0.06		0.29	0.53	0.38	0.03		0.32	0.45
ICC																
	Empty model	0.10	0.01		0.09	0.12	0.10	0.01		0.08	0.13	0.10	0.01		0.09	0.12
	Full model	0.10	0.01		0.09	0.12	0.11	0.01		0.08	0.14	0.10	0.01		0.09	0.12
Wald Chi Square test																
	Chi-Square	178.31					84.32					216.78				
	*p* value	<0.001					<0.001					<0.001				

Note: * Average marginal effects (AME) of location of social health insurance funds: 4.8% (1.8%, 7.7%) for rural-to-urban respondents; 1.9% (−1.8%, 5.6%) for urban-to-urban respondents; 3.5% (1.3%, 5.8%) for all respondents. ^†^ ICC: intraclass correlation coefficient; AOR: adjusted odds ratio; SE: standard error; CI: confidence interval.

## Data Availability

The datasets used in this study are not publicly available due to restrictions imposed on this study, but they are available from the Migrant Population Service Center, National Health Commission of China upon reasonable requests.

## Supplementary Materials

The following are available online at https://www.mdpi.com/1660-4601/17/17/6327/s1, Table S1. Interaction effect of location and type of social health insurance on the establishment of personal health records: results of two-level logistic regression models. Table S2. Interaction effect of location and type of social health insurance on the establishment of personal health records: results of two-level logistic regression models. Table S3. Interaction effect between location and type of social health insurance on visits to local physicians when needed: results of two-level logistic regression models. Table S4. Interaction effect between location and type of social health insurance on visits to local physicians when needed: results of two-level logistic regression models.
