# Supplementary material for: Inequality in Health Services for Internal Migrants in China: A National Cross-Sectional Study on the Role of Fund Location of Social Health Insurance
Source: Int J Environ Res Public Health. 2020 Aug 31;17(17):6327. doi: 10.3390/ijerph17176327 (PMC7504160; doi:10.3390/ijerph17176327)
Supplement: Supplementary file 1 [file ijerph-17-06327-s001.pdf]

Table S1 Interaction effect of location and type of social health insurance on the establishment of personal health records: results of two-level logistic regression models

Variables		Rural to Urban Respondents					Urban to Urban Respondents					All Respondents				
		AOR/ICC	SE	P Value	95%CI		AOR/ICC	SE	P Value	95%CI		AOR/ICC	SE	P Value	95%CI	
Fixed effects (Level 1)																
Enrolment with a local social health insurance program	No (reference)															
	Yes *	1.66	0.11	<0.001	1.46	1.88	1.62	0.23	<0.001	1.23	2.14	1.62	0.08	<0.001	1.46	1.79
Interaction between location and type social health insurance	Without a local BMIUE/RNCMS (reference)															
	Location × BMIUE	0.61	0.10	<0.001	0.44	0.82	0.79	0.16	0.26	0.53	1.19	0.71	0.10	0.01	0.54	0.93
	Location × Others	0.04	0.07	0.04	<0.001	0.84	1.81	1.59	0.50	0.32	10.09	1.27	0.85	0.73	0.34	4.70
Gender	Male (reference)															
	Female	1.21	0.04	<0.001	1.13	1.29	1.19	0.07	<0.001	1.06	1.34	1.21	0.03	<0.001	1.14	1.28
Age (Years)	15–24 (reference)															
	25–34	1.00	0.07	0.95	0.87	1.15	1.10	0.12	0.38	0.89	1.36	1.01	0.07	0.85	0.88	1.16
	35–44	1.04	0.08	0.64	0.90	1.20	1.01	0.12	0.96	0.80	1.27	1.01	0.08	0.86	0.88	1.17
	45–54	0.93	0.07	0.37	0.80	1.09	1.01	0.15	0.96	0.76	1.35	0.94	0.07	0.38	0.81	1.09
	55+	1.09	0.15	0.55	0.83	1.42	1.27	0.19	0.12	0.94	1.71	1.15	0.14	0.24	0.91	1.46
Marital status	Never married/Single (reference)															
	Married	1.30	0.09	<0.001	1.13	1.49	1.30	0.14	0.01	1.06	1.60	1.32	0.09	<0.001	1.14	1.52
	Divorced	1.19	0.15	0.18	0.92	1.53	1.37	0.23	0.07	0.98	1.91	1.25	0.14	0.05	1.00	1.56
	Widowed	1.12	0.22	0.55	0.77	1.64	1.21	0.42	0.59	0.61	2.37	1.12	0.19	0.51	0.80	1.57
Educational attainment	Illiterate (reference)															
	Primary school	1.34	0.14	0.01	1.09	1.64	1.55	0.56	0.23	0.76	3.15	1.39	0.14	<0.001	1.14	1.69
	Junior middle school	1.55	0.16	<0.001	1.27	1.90	1.96	0.69	0.06	0.99	3.91	1.63	0.16	<0.001	1.35	1.98
	Senior middle school	1.84	0.22	<0.001	1.45	2.33	2.08	0.75	0.04	1.03	4.20	1.90	0.22	<0.001	1.52	2.37
	University/college	1.96	0.24	<0.001	1.54	2.49	2.21	0.82	0.03	1.06	4.58	2.02	0.22	<0.001	1.63	2.51
Employment	Unemployed (reference)															
	Employed	0.94	0.04	0.12	0.86	1.02	0.99	0.08	0.91	0.84	1.17	0.94	0.03	0.11	0.88	1.01
Household income ranking	Lowest (<percentile 20, reference)															

	Lower (percentile20–39.9)	1.13	0.05	0.01	1.03	1.24	1.02	0.07	0.79	0.89	1.17	1.11	0.05	0.01	1.02	1.20
	Middle (percentile40–59.9)	1.12	0.05	0.01	1.03	1.23	1.02	0.10	0.81	0.84	1.24	1.10	0.05	0.03	1.01	1.20
	Higher (percentile60–79.9)	1.01	0.05	0.77	0.93	1.11	1.01	0.10	0.90	0.83	1.23	1.02	0.04	0.69	0.93	1.11
	Highest (≥percentile80)	1.11	0.07	0.10	0.98	1.24	0.93	0.10	0.50	0.75	1.15	1.05	0.06	0.36	0.94	1.17
Type of social health insurance	BMIUR/RNCMS (reference)															
	BMIUE	1.60	0.23	<0.001	1.21	2.11	1.17	0.19	0.34	0.85	1.60	1.36	0.16	0.01	1.09	1.71
	Others	17.29	25.35	0.05	0.98	305.98	1.03	0.41	0.93	0.47	2.25	1.31	0.61	0.57	0.52	3.28
Self-rating of health	Good (reference)															
	General	0.75	0.04	<0.001	0.67	0.84	0.66	0.04	<0.001	0.59	0.75	0.73	0.03	<0.001	0.67	0.80
	Poor	0.83	0.08	0.05	0.69	1.00	0.74	0.13	0.08	0.53	1.03	0.80	0.06	0.01	0.69	0.94
Two-week morbidity	No (reference)															
	Yes	1.01	0.09	0.95	0.85	1.20	1.10	0.14	0.42	0.87	1.41	1.04	0.08	0.64	0.89	1.21
Chronic morbidity	No (reference)															
	Yes	1.24	0.07	<0.001	1.11	1.38	1.43	0.17	<0.001	1.13	1.80	1.30	0.06	<0.001	1.18	1.43
Radom effects (Level 2)																
	Variance (enrolment with local social health insurance)	0.58	0.08		0.44	0.75	0.71	0.15		0.46	1.08	0.59	0.12		0.40	0.88
	Variance (intercept)	2.74	0.18		2.42	3.11	1.92	0.17		1.62	2.27	2.55	0.19		2.21	2.94
ICC																
	Empty model	0.45	0.02		0.42	0.48	0.36	0.02		0.32	0.40	0.45	0.01		0.42	0.48
	Full model	0.45	0.02		0.42	0.49	0.37	0.02		0.33	0.41	0.44	0.02		0.40	0.47
Wald Chi Square test																
	Chi-Square			386.9					192.12					432.31		
	p value			<0.001					<0.001					<0.001		

* Average marginal effects (AME) of location of social health insurance funds for BMIUR/RNCMS: 7.7% (5.8%, 9.5%) for rural-to-urban respondents; 8.5% (4.1%, 13.0%) for urban-to-urban respondents; 7.6% (6.0%, 9.2%) for all respondents.

Table S2 Interaction effect of location and type of social health insurance on the establishment of personal health records: results of two-level logistic regression models

Variables		Rural to Urban Respondents					Urban to Urban Respondents					All Respondents				
		AOR/ICC	SE	P Value	95%CI		AOR/ICC	SE	P Value	95%CI		AOR/ICC	SE	P Value	95%CI	
Fixed effects (Level 1)																
Enrolment with a local social health insurance program	No (reference)															
	Yes *	1.00	0.15	0.98	0.75	1.34	1.28	0.21	0.13	0.93	1.76	1.14	0.16	0.33	0.87	1.50
Interaction between location and type social health insurance	Without a local BMIUE/RNCMS (reference)															
	Location × BMIUE	1.65	0.26	<0.001	1.21	2.25	1.27	0.26	0.26	0.84	1.90	1.42	0.20	0.01	1.07	1.87
	Location × Others	0.07	0.11	0.08	0.00	1.41	2.29	2.03	0.35	0.40	13.08	1.79	1.19	0.38	0.49	6.59
Gender	Male (reference)															
	Female	1.21	0.04	<0.001	1.13	1.29	1.19	0.07	<0.01	1.06	1.34	1.21	0.03	<0.001	1.14	1.28
Age (Years)	15–24 (reference)															
	25–34	1.00	0.07	0.95	0.87	1.15	1.10	0.12	0.38	0.89	1.36	1.01	0.07	0.85	0.88	1.16
	35–44	1.04	0.08	0.64	0.90	1.20	1.01	0.12	0.96	0.80	1.27	1.01	0.08	0.86	0.88	1.17
	45–54	0.93	0.07	0.37	0.80	1.09	1.01	0.15	0.96	0.76	1.35	0.94	0.07	0.38	0.81	1.09
	55+	1.09	0.15	0.55	0.83	1.42	1.27	0.19	0.12	0.94	1.71	1.15	0.14	0.24	0.91	1.46
Marital status	Never married/Single (reference)															
	Married	1.30	0.09	<0.001	1.13	1.49	1.30	0.14	0.01	1.06	1.60	1.32	0.09	<0.001	1.14	1.52
	Divorced	1.19	0.15	0.18	0.92	1.53	1.37	0.23	0.07	0.98	1.91	1.25	0.14	0.05	1.00	1.56
	Widowed	1.12	0.22	0.55	0.77	1.64	1.21	0.42	0.59	0.61	2.37	1.12	0.19	0.51	0.80	1.57
Educational attainment	Illiterate (reference)															
	Primary school	1.34	0.14	0.01	1.09	1.64	1.55	0.56	0.23	0.76	3.15	1.39	0.14	<0.001	1.14	1.69
	Junior middle school	1.55	0.16	<0.001	1.27	1.90	1.96	0.69	0.06	0.99	3.91	1.63	0.16	<0.001	1.35	1.98
	Senior middle school	1.84	0.22	<0.001	1.45	2.33	2.08	0.75	0.04	1.03	4.20	1.90	0.22	<0.001	1.52	2.37
	University/college	1.96	0.24	<0.001	1.54	2.49	2.21	0.82	0.03	1.06	4.58	2.02	0.22	<0.001	1.63	2.51
Employment	Unemployed (reference)															
	Employed	0.94	0.04	0.12	0.86	1.02	0.99	0.08	0.91	0.84	1.17	0.94	0.03	0.11	0.88	1.01
Household income ranking	Lowest (<percentile 20, reference)															

	Lower (percentile20–39.9)	1.13	0.05	0.01	1.03	1.24	1.02	0.07	0.79	0.89	1.17	1.11	0.05	0.01	1.02	1.20
	Middle (percentile40–59.9)	1.12	0.05	0.01	1.03	1.23	1.02	0.10	0.81	0.84	1.24	1.10	0.05	0.03	1.01	1.20
	Higher (percentile60–79.9)	1.01	0.05	0.77	0.93	1.11	1.01	0.10	0.90	0.83	1.23	1.02	0.04	0.69	0.93	1.11
	Highest (≥percentile80)	1.11	0.07	0.10	0.98	1.24	0.93	0.10	0.50	0.75	1.15	1.05	0.06	0.36	0.94	1.17
Type of social health insurance	BMIUR/RNCMS (reference)															
	BMIUE	0.63	0.09	<0.001	0.47	0.82	0.86	0.14	0.34	0.62	1.18	0.73	0.08	0.01	0.59	0.92
	Others	10.82	15.92	0.11	0.60	193.76	0.89	0.37	0.77	0.39	2.00	0.96	0.46	0.93	0.38	2.43
Self-rating of health	Good (reference)															
	General	0.75	0.04	<0.001	0.67	0.84	0.66	0.04	<0.001	0.59	0.75	0.73	0.03	<0.001	0.67	0.80
	Poor	0.83	0.08	0.05	0.69	1.00	0.74	0.13	0.08	0.53	1.03	0.80	0.06	0.01	0.69	0.94
Two-week morbidity	No (reference)															
	Yes	1.01	0.09	0.95	0.85	1.20	1.10	0.14	0.42	0.87	1.41	1.04	0.08	0.64	0.89	1.21
Chronic morbidity	No (reference)															
	Yes	1.24	0.07	<0.001	1.11	1.38	1.43	0.17	<0.01	1.13	1.80	1.30	0.06	<0.001	1.18	1.43
Radom effects (Level 2)																
	Variance (enrolment with local social health insurance)	0.58	0.08		0.44	0.75	0.71	0.15		0.46	1.08	0.59	0.12		0.40	0.88
	Variance (intercept)	2.74	0.18		2.42	3.11	1.92	0.17		1.62	2.27	2.55	0.19		2.21	2.94
ICC																
	Empty model	0.45	0.02		0.42	0.48	0.36	0.02		0.32	0.40	0.45	0.01		0.42	0.48
	Full model	0.45	0.02		0.42	0.49	0.37	0.02		0.33	0.41	0.44	0.02		0.40	0.47
Wald Chi Square test																
	Chi-Square	386.9					192.12					432.31				
	p value	<0.001					<0.001					<0.001				

* Average marginal effects (AME) of location of social health insurance funds for BMIUE: 0.3% (-3.9%, 4.5%) for rural-to-urban respondents; 4.7% (-0.3%, 9.7%) for urban-to-urban respondents; 2.3% (-1.7%, 6.3%) for all respondents.

Table S3 Interaction effect between location and type of social health insurance on visits to local physicians when needed: results of two-level logistic regression models

Variables		Rural to Urban Respondents					Urban to Urban Respondents					All Respondents				
		AOR/ICC	SE	P Value	95%CI		AOR/ICC	SE	P Value	95%CI		AOR/ICC	SE	P Value	95%CI	
Fixed effects (Level 1)																
Enrolment with a local social health insurance program	No (reference)															
	Yes *	1.16	0.08	0.04	1.01	1.33	1.00	0.12	0.98	0.79	1.28	1.11	0.07	0.10	0.98	1.25
Interaction between location and type social health insurance	Without a local BMIUE/RNCMS (reference)															
	Location × BMIUE	1.54	0.26	0.01	1.10	2.15	1.15	0.21	0.45	0.80	1.65	1.17	0.14	0.18	0.93	1.48
	Location × Others	14.78	21.58	0.07	0.84	258.72	5.55	4.51	0.04	1.13	27.30	6.75	4.66	0.01	1.74	26.13
Gender	Male (reference)															
	Female	1.06	0.04	0.10	0.99	1.13	1.15	0.07	0.03	1.02	1.30	1.08	0.03	0.01	1.02	1.14
Age (Years)	15–24 (reference)															
	25–34	1.09	0.08	0.22	0.95	1.25	0.90	0.13	0.45	0.68	1.19	1.05	0.06	0.43	0.93	1.18
	35–44	1.05	0.08	0.46	0.91	1.22	0.83	0.13	0.22	0.61	1.12	1.00	0.07	0.99	0.88	1.14
	45–54	0.91	0.07	0.24	0.79	1.06	0.85	0.13	0.30	0.62	1.16	0.89	0.06	0.11	0.78	1.03
	55+	1.02	0.11	0.85	0.82	1.27	0.88	0.17	0.50	0.60	1.28	0.99	0.09	0.93	0.84	1.18
Marital status	Never married/Single (reference)															
	Married	1.03	0.08	0.70	0.89	1.19	1.08	0.13	0.55	0.84	1.37	1.03	0.08	0.71	0.89	1.19
	Divorced	1.04	0.15	0.79	0.79	1.37	1.13	0.28	0.62	0.70	1.83	1.04	0.13	0.76	0.81	1.33
	Widowed	0.90	0.19	0.60	0.60	1.35	1.46	0.41	0.17	0.85	2.52	0.99	0.18	0.97	0.70	1.40
Educational attainment	Illiterate (reference)															
	Primary school	1.05	0.10	0.61	0.87	1.27	0.65	0.19	0.14	0.37	1.15	1.01	0.10	0.90	0.84	1.22
	Junior middle school	1.14	0.11	0.17	0.94	1.38	0.67	0.20	0.18	0.37	1.20	1.10	0.10	0.28	0.93	1.31
	Senior middle school	1.09	0.15	0.51	0.84	1.43	0.74	0.22	0.30	0.41	1.32	1.07	0.12	0.54	0.86	1.33
	University/college	1.12	0.13	0.36	0.88	1.42	0.75	0.23	0.36	0.41	1.38	1.09	0.10	0.39	0.90	1.31
Employment	Unemployed (reference)															
	Employed	0.89	0.04	0.01	0.80	0.98	1.12	0.12	0.29	0.91	1.39	0.93	0.04	0.10	0.86	1.01
Household income ranking	Lowest (<percentile 20, reference)															
	Lower (percentile20–39.9)	1.01	0.05	0.75	0.93	1.11	1.14	0.13	0.25	0.91	1.42	1.03	0.05	0.49	0.95	1.12

	Middle (percentile40–59.9)	1.00	0.06	0.93	0.90	1.12	1.15	0.14	0.24	0.91	1.46	1.03	0.05	0.61	0.93	1.14
	Higher (percentile60–79.9)	1.02	0.05	0.63	0.93	1.13	1.03	0.11	0.78	0.84	1.26	1.01	0.05	0.79	0.92	1.11
	Highest (≥percentile80)	1.07	0.08	0.41	0.92	1.24	0.96	0.12	0.76	0.76	1.22	1.02	0.07	0.82	0.88	1.17
Type of social health insurance	BMIUR/RNCMS (reference)															
	BMIUE	0.59	0.10	<0.001	0.43	0.81	0.90	0.13	0.46	0.69	1.19	0.78	0.07	0.01	0.65	0.93
	Others	2.34	2.22	0.37	0.36	15.02	0.78	0.22	0.38	0.45	1.35	0.82	0.19	0.40	0.52	1.30
Self-rating of health	Good (reference)															
	General	1.15	0.06	0.01	1.04	1.27	1.16	0.11	0.10	0.97	1.39	1.14	0.05	<0.001	1.05	1.25
	Poor	1.81	0.18	<0.001	1.49	2.20	1.88	0.46	0.01	1.17	3.03	1.80	0.18	<0.001	1.47	2.20
Two-week morbidity	No (reference)															
	Yes	1.21	0.06	<0.001	1.10	1.34	1.20	0.11	0.05	1.00	1.44	1.21	0.06	<0.001	1.10	1.32
Chronic morbidity	No (reference)															
	Yes	1.30	0.10	<0.001	1.12	1.51	1.40	0.18	0.01	1.08	1.81	1.33	0.09	<0.001	1.17	1.53
Radom effects (Level 2)																
	Variance (enrolment with local social health insurance)	0.35	0.06		0.25	0.51	0.21	0.07		0.11	0.41	0.25	0.04		0.18	0.35
	Variance (intercept)	0.37	0.04		0.31	0.45	0.39	0.06		0.29	0.53	0.38	0.03		0.32	0.45
ICC																
	Empty model	0.10	0.01		0.09	0.12	0.10	0.01		0.08	0.13	0.10	0.01		0.09	0.12
	Full model	0.10	0.01		0.09	0.12	0.11	0.01		0.08	0.14	0.10	0.01		0.09	0.12
Wald Chi Square test																
	Chi-Square			206.87					97.23					225.23		
	p value			<0.001					<0.001					<0.001		

* Average marginal effects (AME) of location of social health insurance funds for BMIUR/RNCMS: 3.2% (0.1%, 6.3%) for rural-to-urban respondents; 0.1% (-5.3%, 4.9%) for urban-to-urban respondents; 2.2% (-0.5%, 4.9%) for all respondents.

Table S4 Interaction effect between location and type of social health insurance on visits to local physicians when needed: results of two-level logistic regression models

Variables		Rural to Urban Respondents					Urban to Urban Respondents					All Respondents				
		AOR/ICC	SE	P Value	95%CI		AOR/ICC	SE	P Value	95%CI		AOR/ICC	SE	P Value	95%CI	
Fixed effects (Level 1)																
Enrolment with a local social health insurance program	No (reference)															
	Yes *	1.78	0.29	<0.001	1.29	2.46	1.15	0.14	0.26	0.90	1.47	1.30	0.13	0.01	1.07	1.58
Interaction between location and type social health insurance	Without a local BMIUE/RNCMS (reference)															
	Location × BMIUE	0.65	0.11	0.01	0.47	0.91	0.87	0.16	0.45	0.61	1.25	0.85	0.10	0.18	0.68	1.07
	Location × Others	9.60	14.09	0.12	0.54	170.27	4.84	3.85	0.05	1.02	23.05	5.75	3.99	0.01	1.48	22.43
Gender	Male (reference)															
	Female	1.06	0.04	0.10	0.99	1.13	1.15	0.07	0.03	1.02	1.30	1.08	0.03	0.01	1.02	1.14
Age (Years)	15–24 (reference)															
	25–34	1.09	0.08	0.22	0.95	1.25	0.90	0.13	0.45	0.68	1.19	1.05	0.06	0.43	0.93	1.18
	35–44	1.05	0.08	0.46	0.91	1.22	0.83	0.13	0.22	0.61	1.12	1.00	0.07	0.99	0.88	1.14
	45–54	0.91	0.07	0.24	0.79	1.06	0.85	0.13	0.30	0.62	1.16	0.89	0.06	0.11	0.78	1.03
	55+	1.02	0.11	0.85	0.82	1.27	0.88	0.17	0.50	0.60	1.28	0.99	0.09	0.93	0.84	1.18
Marital status	Never married/Single (reference)															
	Married	1.03	0.08	0.70	0.89	1.19	1.08	0.13	0.55	0.84	1.37	1.03	0.08	0.71	0.89	1.19
	Divorced	1.04	0.15	0.79	0.79	1.37	1.13	0.28	0.62	0.70	1.83	1.04	0.13	0.76	0.81	1.33
	Widowed	0.90	0.19	0.60	0.60	1.35	1.46	0.41	0.17	0.85	2.52	0.99	0.18	0.97	0.70	1.40
Educational attainment	Illiterate (reference)															
	Primary school	1.05	0.10	0.61	0.87	1.27	0.65	0.19	0.14	0.37	1.15	1.01	0.10	0.90	0.84	1.22
	Junior middle school	1.14	0.11	0.17	0.94	1.38	0.67	0.20	0.18	0.37	1.20	1.10	0.10	0.28	0.93	1.31
	Senior middle school	1.09	0.15	0.51	0.84	1.43	0.74	0.22	0.30	0.41	1.32	1.07	0.12	0.54	0.86	1.33
	University/college	1.12	0.13	0.36	0.88	1.42	0.75	0.23	0.36	0.41	1.38	1.09	0.10	0.39	0.90	1.31
Employment	Unemployed (reference)															
	Employed	0.89	0.04	0.01	0.80	0.98	1.12	0.12	0.29	0.91	1.39	0.93	0.04	0.10	0.86	1.01
Household income ranking	Lowest (<percentile 20, reference)															

	Lower (percentile20–39.9)	1.01	0.05	0.75	0.93	1.11	1.14	0.13	0.25	0.91	1.42	1.03	0.05	0.49	0.95	1.12
	Middle (percentile40–59.9)	1.00	0.06	0.93	0.90	1.12	1.15	0.14	0.24	0.91	1.46	1.03	0.05	0.61	0.93	1.14
	Higher (percentile60–79.9)	1.02	0.05	0.63	0.93	1.13	1.03	0.11	0.78	0.84	1.26	1.01	0.05	0.79	0.92	1.11
	Highest (≥percentile80)	1.07	0.08	0.41	0.92	1.24	0.96	0.12	0.76	0.76	1.22	1.02	0.07	0.82	0.88	1.17
Type of social health insurance	BMIUR/RNCMS (reference)															
	BMIUE	1.69	0.27	<0.001	1.23	2.33	1.11	0.15	0.46	0.84	1.46	1.28	0.11	0.01	1.08	1.53
	Others	3.96	3.82	0.15	0.60	26.22	0.87	0.21	0.56	0.53	1.40	1.05	0.24	0.83	0.67	1.64
Self-rating of health	Good (reference)															
	General	1.15	0.06	0.01	1.04	1.27	1.16	0.11	0.10	0.97	1.39	1.14	0.05	<0.001	1.05	1.25
	Poor	1.81	0.18	<0.001	1.49	2.20	1.88	0.46	0.01	1.17	3.03	1.80	0.18	<0.001	1.47	2.20
Two-week morbidity	No (reference)															
	Yes	1.21	0.06	<0.001	1.10	1.34	1.20	0.11	0.05	1.00	1.44	1.21	0.06	<0.001	1.10	1.32
Chronic morbidity	No (reference)															
	Yes	1.30	0.10	<0.001	1.12	1.51	1.40	0.18	0.01	1.08	1.81	1.33	0.09	<0.001	1.17	1.53
Radom effects (Level 2)																
	Variance (enrolment with local social health insurance)	0.35	0.06		0.25	0.51	0.21	0.07		0.11	0.41	0.25	0.04		0.18	0.35
	Variance (intercept)	0.37	0.04		0.31	0.45	0.39	0.06		0.29	0.53	0.38	0.03		0.32	0.45
ICC																
	Empty model	0.10	0.01		0.09	0.12	0.10	0.01		0.08	0.13	0.10	0.01		0.09	0.12
	Full model	0.10	0.01		0.09	0.12	0.11	0.01		0.08	0.14	0.10	0.01		0.09	0.12
Wald Chi Square test																
	Chi-Square			206.87					97.23					225.23		
	p value			<0.001					<0.001					<0.001		

* Average marginal effects (AME) of location of social health insurance funds at BMIUE: 12.8% (5.6%, 20.0%) for rural-to-urban respondents; 3.1% (-2.3%, 8.6%) for urban-to-urban respondents; 5.8% (1.4%, 10.1%) for all respondents.
